# Who Was Concerned about Radiation, Food Safety, and Natural Disasters after the Great East Japan Earthquake and Fukushima Catastrophe? A Nationwide Cross-Sectional Survey in 2012

**DOI:** 10.1371/journal.pone.0106377

**Published:** 2014-09-02

**Authors:** Takashi Sugimoto, Tomohiro Shinozaki, Takashi Naruse, Yuki Miyamoto

**Affiliations:** 1 Department of Health Technology Assessment and Public Policy, Graduate School of Public Policy, The University of Tokyo, Bunkyo, Tokyo, Japan; 2 Department of Biostatistics, Graduate School of Medicine, The University of Tokyo, Bunkyo, Tokyo, Japan; 3 Department of Community Health Nursing, Graduate School of Medicine, The University of Tokyo, Bunkyo, Tokyo, Japan; 4 Department of Psychiatric Nursing, Graduate School of Medicine, The University of Tokyo, Bunkyo, Tokyo, Japan; University of Jyväskylä, Finland

## Abstract

**Background:**

Disaster-related concerns by sub-populations have not been clarified after the great East Japan earthquake and the Fukushima nuclear power plant incidents. This paper assesses who was concerned about radiation, food safety, and natural disasters among the general population in order to buffer such concerns effectively.

**Methods:**

The hypothesis that women, parents, and family caregivers were most concerned about radiation, food safety, and natural disaster was tested using a varying-intercept multivariable logistic regression with 5809 responses from a nationwide cross-sectional survey random-sampled in March 2012.

**Results:**

Many people were at least occasionally concerned about radiation (53.5%), food safety (47.3%), and about natural disaster (69.5%). Women were more concerned than men about radiation (OR = 1.67; 95% CI = 1.35–2.06), food safety (1.70; 1.38–2.10), and natural disasters (1.74; 1.39–2.19). Parents and family care needs were not significant. Married couples were more concerned about radiation (1.53; 1.33–1.77), food safety (1.38; 1.20–1.59), and natural disasters (1.30; 1.12–1.52). Age, child-cohabitation, college-completion, retirement status, homemaker status, and the house-damage certificate of the last disaster were also associated with at least one concern. Participants from the Kanto region were more concerned about radiation (2.08; 1.58–2.74) and food safety (1.30; 1.07–1.59), which demonstrate similar positive associations to participants from Tohoku where a disaster relief act was invoked (3.36; 2.25–5.01 about radiation, 1.49; 1.08–2.06 about food safety).

**Conclusions:**

Sectioning the populations by gender and other demographics will clarify prospective targets for interventions, allow for a better understanding of post-disaster concerns, and help communicate relevant information effectively.

## Introduction

The great East Japan earthquake on March 11, 2011, subsequent tsunamis, and the Fukushima nuclear power plant (NPP) accident had a large impact on the Japanese population [1]. The Japanese prime minister made a bold assertion to the world that the Fukushima crisis was under control during his presentation to the international Olympic committee in September 2013 [2] [3]. In that same month, however, the editorial “Nuclear Error” in *Nature* persuaded Japan to tackle the Fukushima issues with international cooperation because “few independent measurements of radiation exposure are available, and it is worryingly unclear how these leaks might affect human health, the environment and food safety” [4]. The editorial was metaphrased in Japanese and rapidly spread via Twitter or Facebook [5]. It takes several years to write and publish scientific reports on internal contamination as a result of the Fukushima NPP [6] [7] [8] [9]. Hence, some people may have felt a lack of direct evidence of safety across Japan [10] [11], especially those who doubted the authenticity of monitoring and evaluation by the government. After the disaster, some doubted the competence of the Japanese government to communicate relevant evidence to the public [4] [10]. These doubts seem to have fueled long-term disaster-related concerns among the general population.

Although holding such concerns can seem less relevant compared to diagnosed diseases, concerns can be a cause of physical and mental health problems. For example, some Japanese have evacuated their homes to be further away from the Fukushima NPP. Evacuation has been reported as a risk factor for mortality [12] or subjective health [1] perhaps via physical strain, economic strain, and disrupted social support. Another plausible cause of physical and mental health problems is food avoidance, which if excessive could result in malnutrition or distress. Furthermore, people who think they may have been exposed to radiation may feel stigmatized, including self-stigma, and conflict between family members may arise if people have different opinions on health risks [13]. Such disaster-related concerns likely vary across different sub-populations. To identify sub-populations that are particularly concerned about health risks is of major importance to designing effective interventions that might include public information campaigns for buffering people concerns.

Across disasters, risk factors for psychological consequences include the severity of the disaster and personal characteristics, such as being a woman, or having children [14] [15] [16]. Mothers of young children reported high levels of psychological distress after the Chernobyl accident, primarily because of concerns about adverse health effects for their children and future generations [17]. The study, however, only included women with children so these results may not be generalizable to women without children, or to men [17]. In addition to parenthood, disaster-related concerns may also be elevated in people living with family members who need medical care or daily activity assistance. Families with people with diseases or disabilities may be as sensitive to disasters as people with children.

Geographic location can also be a critical factor. Regional differences in the anticipation of future disaster risks have been reported in university students [18] and in subjective health among youth [1]. Although some reports have investigated disaster-related concerns in the disaster-struck area [11] [19], few studies have reported on disaster-related concerns that have spread among the general population across Japan after the great East Japan disaster.

The aim of the present study was to assess who was more concerned about radiation, food safety, and future natural disasters among the general population of Japan. We hypothesized that being a woman and a parent, as well as living with family members with care needs, would be associated with higher levels of concerns.

## Methods

### Dataset Acquisition and Ethical Considerations

The dataset for this secondary analysis, from the survey on quality of life in 2012 (Seikatsu no sitsu ni kansuru chosa (in Japanese); No. 0856), was provided by the Social Science Japan Data Archive maintained by the Centre for Social Research and Data Archives at the University of Tokyo. The survey was conducted March 1–16, 2012, to investigate quality of life and its associated factors, by the Economic and Social Research Institute, Cabinet Office, of the Government of Japan. The survey was conducted according to ethical guidelines for social science research. We did not apply for research approval from an ethics committee because this analysis used a de-identified dataset edited and offered by the Social Science Japan Data Archive to academic researchers and students without any ethical or financial requests [1].

### Participants

The survey randomly recruited 10440 people 15-years-old and older, stratified into three levels (city, area, and household) based on a national basic resident register in Japan. The self-administered questionnaires were placed at each selected household and collected via a subsequent direct visit to each household. The response rate was 61.8% and we obtained a dataset with 6451 participants. We excluded participants with missing values in reported concerns (39 units about radiation, 37 units about food safety, 35 units about natural disaster), parenthood (64 units), child cohabitation (13 units, conditional on having a child/children), marital status (14 units), family care needs (124 units), education (41 units), employment status (112 units) and household income (313 units). Consequently, 5809 complete cases (55.6% of recruited people and 90.0% of participants) remained for our analysis. Regarding the item-non-response, we did a sensitivity analysis using multiple imputation as explained below. Regarding the unit-non-response, we confirmed no large differences in marital status, gender, and 5-year age groups from that of the census in 2010 [20]. Briefly, our sample had fewer people below 40-years-old. Compared with the national survey of family income and expenditure in 2009, the unit-response included several more percentages of people with household income levels below 3 million yen [20].

### Outcomes: Concern about radiation, food safety, and natural disasters

Participants were asked how much they were concerned about three issues: Radiation, food safety, and natural disasters. Participants could respond using a set of five answer choices: Always, Occasionally, Indifferent (in Japanese, this category would mean “No opinion” or “I cannot answer”), Rarely, or Never. The question was not psychometrically validated. We did not seek to aggregate these three concerns because they differ in concept and we believed it most useful to assess them separately. Correlations are provided in the result section.

### Personal-level Independent variables

Gender (reference: Man) and parental status were included as predictor variables. Marital status and child cohabitation were considered to be potential confounders of parenthood and outcomes. We also included whether participants live with family members with care needs as a predictor variable. Persons living in the same house with family members with care needs were scored ‘1’ and persons not living with such family members were scored ‘0’. We assumed care needs when a family member was either bedridden, on medical leave, had a disability, and had a certification from the Japanese long-term care insurance system for frail adults [21]. Education status referred to completing a college degree or not. Dummy variables for employment status were constructed as follows: student, retired, homemaker, others (including people seeking a job), and a reference for people with income from work (including regularly-employed, part-time worker, self-owned business worker, and side-job worker). Household income levels were as follows: 0 = no household income, 1 = less than 0.9 million yen, 2 = 1.0–1.9 million yen, 3 = 2.0–2.9 million yen, 4 = 3.0–4.9 million yen, 5 = 5.0–6.9 million yen, 6 = 7.0–9.9 million yen, 7 = at least 10 million yen.

The extent of damage from the great East Japan earthquake was considered to be an essential variable. The available measures of extent of damage were the current evacuation status of a respondent or their family and an official house-damage certificate from the last disaster. House-damage certificates were provided after on-site investigations by municipal officers reporting the extent of household damage. Damage included partial destruction from earthquake shocks, tsunamis, fires, or floods. A disaster-victim certificate was required to apply for insurance claims and some programmes for disaster victims in Japan.

### Regional-level independent variables

Four regions were introduced according to invocation of the disaster relief act (DRA) and location: Tohoku-DRA, Tohoku-not-DRA, Kanto, and Others. Briefly, the Tohoku-DRA region houses the Fukushima NPP and was heavily damaged by tsunami [22]. Tohoku-not-DRA region refers to the western part of Tohoku. Others regions included seven areas: the north island (Hokkaido) and the central-to-western part of Japan (Tokai, Hokuriku, Kinki, Tyugoku, Shikoku, and Kyusyu-Okinawa regions).

### Statistical Analysis

An overview of demographic characteristics was provided by Tohoku-DRA, Tohoku-not-DRA, Kanto, and other areas. Because we used 5809 complete cases, demographics from the 642 respondents with item-non-response were summarized to check that they varied randomly from the sampled population, rather than systematically across regions. Distributions of the results of the three concern questions were separately shown and Spearman's correlations between them were calculated.

Inputs in the logistic regression model were pre-specified according to the research question and published academic literature. Predictors of interest were gender, parenthood, and family care needs. We included interaction terms among the three predictors in the regression model. Adjusted variables (fixed-effect variables) were age, marital status, child cohabitation, college completion, dummy variables for employment status, household income, house-damage certificates from the last disaster, current evacuation status, and dummy variables for the four regions. We incorporated random intercepts of indicators for 10 areas (Tohoku-DRA, Tohoku-not-DRA, Kanto, and the 7 additional areas) to account for unobserved heterogeneity in the “others” region, in addition to the fixed effect of personal-level covariates and the dummy variables for the four regional indicators.

We considered logistic models for ordinal responses (for example, proportional odds models) because the outcome variables (level of concerns) were originally measured in 5-level categories. First, we checked the proportional odds assumption by fitting a series of binary logistic regression models with all potential thresholds (two to four thresholds) to test whether threshold specific odds ratios were homogeneous. Then, we compared two models with and without proportional odds assumptions (an ordinal model and a multinomial logistic model) using a likelihood ratio test [23]. All likelihood ratio statistics for the proportional odds assumption showed *p*<.05. No eligible thresholds for ordinal models were found among the three outcomes. We chose the threshold to be between “occasionally” and “indifferent” because binned residual plots were well balanced around zero for all fitted values and almost within 2 standard errors, suggesting a reasonable model fit [24] [25]. Results were summarized as crude and multivariable adjusted odds ratios (ORs) for each concern, their 95% confidence intervals (CIs), and their *p*-values.

In the iterative regressions for imputation purposes [25], the fitted formula assumed linearity between an outcome with item-non-responses (please see Table 1) and additive predictors. No interaction term was constructed. The predictors were imputed variables, except for an outcome to be imputed, and observed variables without item-non-responses. Although some auxiliary variables were available from the dataset, they were not included into the fitted formula because convergence had hardly been achieved based on the Gelman-Rubin convergence statistic (R-hat) [26]. After convergence by 637 iterations, the inferences were pooled across 3 datasets [25] [26] and presented in Appendix S1 in File S1.

**Table 1:** Demographics of complete cases, excluding respondents with missing data (N = 6451).

	Complete-cases by region					
Variables	All	Tohoku-DRA region	Tohoku-not-DRA region	Kanto region	Other region	Excluded
	(n = 5809)	(*n* = 315)	(*n* = 254)	(*n* = 1465)	(*n* = 3775)	(*n* = 642)
Women (%)	3020 (52.0)	170 (54.0)	143 (56.3)	728 (49.7)	1979 (52.4)	397 (61.8)
Parents (%)	4119 (70.9)	235 (74.6)	192 (75.6)	976 (66.6)	2716 (71.9)	387 (67.0), 64 missing
Family Care needs (%)	739 (12.7)	39 (12.4)	24 (9.4)	173 (11.8)	503 (13.3)	50 (9.7), 124 missing
Age (SD)	51.9 (18.0)	52.7 (17.0)	53.1 (16.9)	51.0 (18.1)	52.2 (18.1)	56.3 (21.3)
Currently married (%)	3557 (61.2)	198 (62.9)	154 (60.6)	881 (60.1)	2324 (61.6)	261 (41.6), 14 missing
Child cohabitation (%)	2480 (42.7)	150 (47.6)	116 (45.7)	615 (42.0)	1599 (42.4)	211 (33.5), 13 missing
College completion (%)	1180 (20.3)	53 (16.8)	33 (13.0)	400 (27.3)	694 (18.4)	59 (9.8), 41 missing
Household income level; mean (SD), median ^†^	3.97 (1.61), 4	3.87 (1.60), 4	3.78 (1.61), 4	4.23 (1.63), 4	3.89 (1.59), 4	3.19 (1.72), 3, 313 missing
Have Job Income (%)	3651 (62.9)	195 (61.9)	147 (57.9)	936 (63.9)	2373 (62.9)	261 (46.1), 76 missing
Homemaker (%)	636 (10.9)	38 (12.1)	28 (11.0)	167 (11.4)	403 (10.7)	55 (10.4), 112 missing
Retired (%)	906 (15.6)	51 (16.2)	48 (18.9)	202 (13.8)	605 (16.0)	106 (20.0), 112 missing
Student (%)	235 (4.0)	11 (3.5)	7 (2.8)	66 (4.5)	151 (4.0)	55 (10.4), 112 missing
Other employment status (%)	381 (6.6)	20 (6.3)	24 (9,.4)	94 (6.4)	243 (6.4)	53 (10.0), 112 missing
House-damage-certificate (%)	310 (5.3)	170 (54.0)	17 (6.7)	99 (6.8)	24 (0.6)	33 (5.1)
Currently evacuated (%)	23 (0.4)	10 (3.2)	2 (0.8)	5 (0.3)	6 (0.2)	5 (0.8)

DRA: disaster relief act. ^†^Range is from level 0 indicating zero household income to level 7 indicating at least 10 million yen.

All the statistical and graphical analyses were conducted using the statistical software R, version 3.0.3 for Windows [27]. Primary functions used were vglm [28], glmer [29], mi, and glmer.mi [26].

## Results

Table 1 shows that respondents with item-non-responses were more likely to be women, less likely to be currently married, less likely to have completed college, and have less household income. Respondents with item-non-response were not selectively distributed among those holding the house-damage certificate and the current evacuation status. Among the four regions, the Kanto region had fewer parents and married participants, more college completion, and higher household income. About half of those in the Tohoku-DRA region had the house-damage certificate, although several percentages of respondents in the Tohoku-not-DRA and Kanto regions also had the certificate.

The distribution of concerns over the five-level categories (Table 2) indicate that about half of the sample were at least occasionally concerned about radiation (53.5%; always 21.8%, occasionally 32.7%), and food safety (47.3%; always 14.8%, occasionally 32.4%), and 69.5% were at least occasionally concerned about natural disaster (always 31.7%, occasionally 37.8%). Table 3 shows a moderate correlation between responses for each possible pair of concerns.

**Table 2:** Distribution of concern about radiation, food safety, and natural disasters by answer category (N = 5809).

	Always	Occasionally	Indifferent	Rarely	Never
Concern about Radiation (%, 95% CI)	1269 (21.8, 20.8–22.9)	1839 (31.7, 30.5–32.8)	1135 (19.5, 18.5–20.5)	906 (15.6, 14.7–16.5)	660 (11.4, 10.6–12.2)
Concern about Food Safety (%, 95% CI)	862 (14.8, 13.9–15.7)	1885 (32.4, 31.2–33.7)	1363 (23.5, 22.4–24.6)	1231 (21.2, 20.1–22.2)	468 (8.1, 7.4–8.8)
Concern about Natural Disaster (%, 95% CI)	1839 (31.7, 30.4–32.8)	2198 (37.8, 36.6–39.1)	915 (15.7, 14.8–16.7)	633 (10.9, 10.1–11.8)	224 (3.9, 3.4–4.4)

Participants were asked how much they were concerned about three areas: Radiation, food safety, and natural disasters. Participants could respond using a set of five answer choices: always, occasionally, indifferent (in Japanese, this category would mean “no opinion” or “I cannot answer”), rarely, or never. 483 responses said “always” to all 3 concerns. The 95% confidence intervals were constructed by bootstrap (10000 times replicates, n = 5809).

**Table 3:** Correlations among concerns about radiation, food safety, and natural disasters (N = 5809).

	Concern about Radiation	Concern about Food Safety	Concern about Natural Disaster
Concern about Radiation	—	0.56	0.61
Concern about Food	0.46	—	0.48
Concern about Natural	0.52	0.39	—

The upper-right shows rank correlation coefficients from five responses to each concern, whilst the lower-left shows correlation coefficients from dichotomized concerns. In the dichotomized concern, the reference category was a newly created category including ‘indifferent,’ ‘never,’ and ‘rarely.’ Concern about radiation: always + occasionally  = 3108; concern about food safety: always + occasionally  = 2747; concern about natural disaster: always + occasionally  = 4037.

Table 4 provides associations between predictors and concerns about radiation, food safety, and natural disasters from logistic models. The main effects of gender, parenthood, and care needs are of primary interest, because all the odds ratios for interaction terms were not significant in multivariable adjusted models. As hypothesized, being a woman was positively associated with all concerns (OR = 1.67; 95% CI = 1.35–2.06 about radiation, 1.70; 1.38–2.10 about food safety, 1.74; 1.39–2.19 about natural disaster). In contrast, the main effects of parenthood and family care needs were not significant in multivariable adjusted models. Looking at personal-level adjusting variables, married people show positive associations with each concern (1.53; 1.33–1.77 about radiation, 1.38; 1.20–1.59 about food safety, 1.30; 1.12–1.52 about natural disaster). Child cohabitation, college completion, and being a homemaker were only associated with concerns about food safety; that means that people living with their child, college graduates, and homemakers were more concerned about food safety than participants who did not live with their child (1.36; 1.18–1.57), had not completed college (1.26; 1.09–1.45), and had an income from work (1.27; 1.05–1.54). Students had fewer concerns about radiation (0.72; 0.53–0.98) and natural disasters (0.67; 0.49–0.92) than people who worked for an income. Retired people and the young had higher levels of concern about food safety and natural disasters. Respondents with house-damage certificates were only concerned about radiation (1.37; 1.00–1.87). Resondents in the Tohoku-DRA region showed positive associations with concern about radiation (3.36; 2.25–5.01) and food safety (1.49; 1.08–2.06). Participants living in the Kanto region also demonstrated concerns about radiation (2.08; 1.58–2.74) and food safety (1.30; 1.07–1.59). Current evacuation status had wider confidence intervals because of the relatively small number of observations in this population. A regression model using imputed datasets yielded essentially the same results (see Appendix S1 in File S1).

**Table 4:** Factors associated with concern about radiation, food Safety, and natural disasters: Multi-level logistic regression analysis (N = 5809).

	Concern about Radiation				Concern about Food Safety				Concern about Natural Disaster			
Predictor variables	Crude OR (95% CI)	*p*-value	Adjusted OR (95% CI)	*p*-value	Crude OR (95% CI)	*p*-value	Adjusted OR (95% CI)	*p*-value	Crude OR (95% CI)	*p*-value	Adjusted OR (95% CI)	*p*-value
Gender (ref: man)	1.70 (1.53–1.89)	<0.001	1.67 (1.35–2.06)	<0.001	1.92 (1.72–2.13)	<0.001	1.70 (1.38–2.10)	<0.001	1.85 (1.65–2.08)	<0.001	1.74 (1.39–2.19)	<0.001
Parenthood (ref: without children)	1.21 (1.08–1.35)	<0.001	0.86 (0.68–1.08)	0.20	1.34 (1.19–1.50)	<0.001	0.92 (0.73–1.16)	0.47	1.00 (0.88–1.13)	0.96	0.87 (0.69–1.11)	0.26
Care Needs (ref: no care needs)	1.16 (0.99–1.36)	0.06	1.47 (0.96–2.26)	0.08	1.07 (0.91–1.25)	0.40	1.25 (0.82–1.91)	0.31	1.03 (0.87–1.21)	0.77	1.33 (0.84–2.11)	0.23
Gender * Parenthood	1.64 (1.47–1.83)	<0.001	1.09 (0.84–1.40)	0.53	1.93 (1.73–2.15)	<0.001	1.16 (0.90–1.49)	0.27	1.65 (1.46–1.86)	<0.001	1.15 (0.87–1.51)	0.33
Gender * Care needs	1.50 (1.21–1.86)	<0.001	0.74 (0.40–1.36)	0.33	1.58 (1.28–1.95)	<0.001	0.75 (0.42–1.37)	0.34	1.45 (1.14–1.85)	0.002	0.73 (0.37–1.43)	0.36
Care needs * Parenthood	1.18 (0.99–1.42)	0.07	0.74 (0.44–1.21)	0.22	1.15 (0.96–1.37)	0.12	0.70 (0.42–1.16)	0.17	0.98 (0.81–1.19)	0.86	0.70 (0.41–1.20)	0.20
Gender * Parenthood * Care needs	1.60 (1.24–2.05)	<0.001	1.43 (0.70–2.94)	0.33	1.84 (1.44–2.35)	<0.001	1.83 (0.90–3.73)	0.10	1.49 (1.13–1.98)	0.005	1.58 (0.72–3.47)	0.25
Age (10 years incr.)	1.00 (0.97–1.03)	0.76	0.96 (0.92–1.22)	0.08	0.96 (0.93–0.98)	0.002	0.92 (0.88–0.96)	<0.001	0.94 (0.91–0.97)	<0.001	0.90 (0.85–0.94)	<0.001
Married	1.40 (1.26–1.56)	<0.001	1.53 (1.33–1.77)	<0.001	1.40 (1.26–1.56)	<0.001	1.38 (1.20–1.59)	<0.001	1.12 (1.00–1.26)	0.046	1.30 (1.12–1.52)	<0.001
Child cohabitation	1.23 (1.11–1.37)	<0.001	1.05 (0.91–1.22)	0.47	1.53 (1.38–1.70)	<0.001	1.36 (1.18–1.57)	<0.001	1.14 (1.01–1.27)	0.03	1.05 (0.90–1.22)	0.57
College Completion	0.91 (0.80–1.04)	0.17	1.00 (0.86–1.15)	0.97	1.05 (0.92–1.19)	0.46	1.26 (1.09–1.45)	0.002	0.95 (0.83–1.09)	0.48	1.06 (0.91–1.24)	0.45
Household income (one level incr.)†	1.01 (0.98–1.05)	0.51	0.99 (0.95–1.39)	0.52	1.00 (0.98–1.05)	0.41	0.97 (0.94–1.01)	0.14	1.00 (0.97–1.04)	0.89	0.97 (0.93–1.02)	0.21
Student (%), ref: with job income	0.66 (0.50–0.86)	0.002	0.72 (0.53–0.98)	0.04	0.73 (0.56–0.96)	0.02	0.89 (0.66–1.21)	0.46	0.81 (0.62–1.07)	0.14	0.67 (0.49–0.92)	0.01
Retired (%), ref: with job income	0.98 (0.85–1.14)	0.83	1.15 (0.95–1.39)	0.14	0.87 (0.76–1.01)	0.07	1.26 (1.05–1.52)	0.01	0.89 (0.76–1.04)	0.13	1.22 (1.00–1.49)	0.047
Homemaker (%), ref: with job income	1.54 (1.30–1.83)	<0.001	1.04 (0.85–1.26)	0.70	1.91 (1.61–2.26)	<0.001	1.27 (1.05–1.54)	0.01	1.42 (1.17–1.72)	<0.001	0.91 (0.73–1.13)	0.39
Other employment status (%), ref: with job income	0.76 (0.61–0.94)	0.01	0.75 (0.60–0.94)	0.01	1.11 (0.90–1.37)	0.31	1.16 (0.93–1.45)	0.20	0.84 (0.67–1.05)	0.12	0.76 (0.60–0.97)	0.03
House Damage Certificate	1.57 (1.16–2.13)	0.004	1.37 (1.00–1.87)	0.049	1.37 (1.05–1.78)	0.02	1.17 (0.88–1.56)	0.27	1.37 (1.00–1.88)	0.05	1.22 (0.87–1.70)	0.25
Current Evacuation	2.83 (0.94–8.52)	0.65	2.61 (0.85–7.99)	0.09	1.28 (0.55–2.95)	0.56	1.27 (0.53–3.01)	0.59	1.77 (0.60–5.29)	0.30	1.65 (0.54–4.99)	0.38
Tohoku-DRA region (ref: Other region)	3.53 (1.93–6.42)	<0.001	3.36 (2.25–5.01)	<0.001	1.60 (1.11–2.30)	0.01	1.49 (1.08–2.06)	0.01	1.84 (1.06–3.20)	0.03	1.67 (0.98–2.83)	0.06
Tohoku-not-DRA region (ref: Other region)	0.91 (0.35–2.33)	0.84	1.11 (0.78–1.59)	0.56	0.84 (0.54–1.32)	0.45	0.86 (0.64–1.18)	0.35	0.63 (0.35–1.14)	0.13	0.67 (0.41–1.10)	0.11
Kanto region (ref: Other region)	1.72 (0.74–4.00)	0.21	2.08 (1.58–2.74)	<0.001	1.24 (0.86–1.79)	0.24	1.30 (1.07–1.59)	0.009	1.34 (0.76–2.36)	0.32	1.39 (0.90–2.15)	0.14

† Range is from level 0 indicating zero household income to level 7 indicating at least 10 million yen. OR: odds ratio. DRA: invoke of the disaster relief act.

## Discussion

### Principal Findings

Many Japanese people were at least occasionally concerned about radiation (53.5%), food safety (47.3%), and natural disasters (69.5%) one year after the great East Japan earthquake and the Fukushima nuclear power plant incident. The hypothesis that women, parents, and people with family care needs would have more concerns about radiation, food safety, and future natural disasters was tested adjusting for potential confounding covariates. Women demonstrated more concerns than men, but parenthood and family care needs were not significant predictors of these concerns. Sub-populations who hold particularly strong concerns about radiation are married women, people holding a home-damage certificate from the last disaster, people living in the Tohoku region with the invocation of the disaster relief act or the Kanto region. Sub-populations who hold particularly strong concerns about food safety are married women, retired people, youth, families living with a child, being college graduate, being a homemaker, people living in the Tohoku region with the invocation of the disaster relief act or the Kanto region. Sub-populations who hold particularly strong concerns about natural disaster are married women, retired people, and youth. The government and municipals can take measures to better understand the concerns across sub-populations and communicate relevant information effectively.

### Comparison with Other Studies

Our report, for the first time, provides the frequency of concerns about radiation, food safety, and natural disasters among a random sample of the general population of Japan after the great East Japan disaster. Compared with the post-Chernobyl report that only included women with children [17], our report includes men and women and people with and without children to test for the relationship between gender, parental status, and the outcome measure of concern. Being a woman seems to be a robust risk factor for radiation-specific concerns across Japan, and not just in the immediate area of the Fukushima NPP [16]. Our findings are in line with other research demonstrating that being a woman was associated with adverse psychological consequences post-disaster [14] [15], although concerns in the present study may not lead to adverse psychological consequences. Those living in the Kanto region or the Tohoku-DRA invoked region also had more concerns about radiation. This finding is consistent with several reports showing that people in the Kanto region had risk perceptions similar to people in a city of the Tohoku region [18], and the Kanto region was the second highest subjective ill-health area following the Tohoku region [1]. Interestingly, marital status was positively associated with higher levels of concerns in the present study, whereas in other literature, marital status is often negatively associated with mental ill-health post-disaster [30]. We analysed the married subgroup of the sample to account for collinearity between being married and parenthood, or between being married and having family care needs. Parenthood and family care needs were not significantly associated with increased concerns (please see Appendix S2 in File S1). Hence, being married may not be a proxy of parenthood or living with family members who needed care giving. Although the mechanism remains unclear, the result that being married could enhance disaster-related concerns is new and could contribute to intervention planning.

### Strengths

First, in our study, the large number of participants was randomly sampled. Although many people have had the opportunity to voice their concerns on Twitter, news media, or in meetings [19], not everyone has these access to these outlets. People's ability to speak out may be limited because of conflict of interest, or a lack of access to information or technology among the seniors. Hence, publicly expressed concerns could suffer from selection bias, resulting in under- or over-estimates of actual public sentiments. Results from the random sample presented in this study are better than information from Twitter.

Second, we could investigate associated factors and potential confounding covariates simultaneously. Furthermore, we used variables of disaster-experience. Because serious house damage was reported to be an associated factor that impairs psychological recovery [31], the house-damage certificate as well as sub-categorization by the DRA was a better than some gross definitions of affected areas, for example “Tohoku region”.

Third, the full survey included questions about many aspects of the quality of life. The disaster-related concern questions were asked in the middle of the questionnaire, so we know that people who were highly interested in the Fukushima issues did not selectively respond to the survey.

Lastly, the item-non-response and the unit-non-response did not cause a great extent of bias. Sensitivity analysis by multiple imputation (Appendix S1 in File S1) yielded associations in line with complete-case analysis in Table 4. Although we could not obtain joint distributions, the comparison with the census indicates no large differences in marginal distributions of some basic demographic variables (gender, age, marital status). The 6451 response units included larger number of 40-year-old and older people compared to the census [20]. Sub-group analyses show that the older sub-group (40-years-old and older) had larger coefficients associating being a woman and being married with higher level of concerns (please see Appendix S3 in File S1) than complete-case analysis of all age ranges (Table 4). This degree of overestimation seems small. Another sub-group analysis by household income of 3 million yen reveals that the higher household income group (at least 3 million yen) had slightly larger coefficients associating being a woman and being married (please see Appendix S4 in File S1). In this case, overestimation shall not be probable. In these sub-group analyses, hypothesized variables showed essentially the same associations and the results of hypothesis testing hold robustly.

### Limitations

Because the outcomes in the present study were not validated measures, we are sceptical as to whether the present study would be more meaningful if a validated general anxiety scale that is not post-disaster specific had been used. The cut-off point or other standard analytical procedures for such a published scale would not be applied to post-disaster settings blindly because responses from many sectors of the population could change during post-disaster uncertainty as discussed in other studies [32] [33]. Although some screening instruments were investigated for validity in post-disaster settings among earthquake survivors [33], validated scales for disaster-related concerns were not available.

Second, the lack of specificity of the three concerns is also a limitation. The participants could have interpreted the questions about concerns over radiation with the Fukushima incidents in mind, or, participants could have interpreted the question to include other types of radiation, for example that from CT scans. Similarly, concerns about food safety could include chemicals or gene recombination, regardless of the great East Japan disaster. Other disasters may also affect the concern about natural disasters, such as typhoons, landslides, floods, or localized torrential downpours. Whereas other factors may play a role, we can deem that the three concerns studied be primarily affected by events stemming from the great East Japan disaster.

Third, the survey period included the first anniversary date of the disaster, March 11, 2012, which could heighten concerns stimulated by broadcast programmes or personal remembrances.

Fourth, feelings of personal vulnerability for families with children may vary according to the children’s age. In a post-Chernobyl report, mother's ages ranged from 28 to 55 years old with the median at 37 [17]. In the present study, mother's age was not included in the analysis, however, we expect the mean age to be higher in this sample. As a result, parenthood is not limited to young children, and parents of adults may have different feelings compared with parents of young children. We included the child cohabitation variable as a proxy of the children's age because the age was not available in the present study. This method may be more useful than dividing the sample into parents of young versus old children because it is arbitrary to determine a threshold without measure of children's age. Also, Japanese parents may be concerned about adverse health effects on their child regardless of that child's age. Possibly, the concerns in the present study may include concerns for grandchildren.

A final limitation is based on the interpretation of evacuation status. At one year post-disaster, relocated people may or may not report themselves as evacuated, given that they have been away from their original homes for so long.

### Implications

One year after the great East Japan disaster, a lot of people are still concerned about radiation, food safety, and natural disasters, especially in the sub-populations mentioned above. The government and municipals may first take measures to better understand the concerns of the general and sub-populations, and subsequently provide relevant information to the public [4] [9] [10], hopefully based on rigorous and quality research [34]. For example, a public campaign for concern about food safety in the Kanto region may particularly aim to target younger women who are married, families living with a child, college graduates, retired people, and homemakers. The difference found in associated factors between the three concerns implies that interventions such as public campaigns, consultations by municipals, and public meetings providing relevant information can target different sub-populations for each type of concern.

## Supporting Information

File S1 **This file contains Appendix S1–Appendix S4.** Appendix S1. Factors associated with concern about radiation, food safety and natural disasters: Pooled inference of multi-level logistic regression analysis after multiple iterative regression imputation (N = 6451). Appendix S2. Factors associated with concern about radiation, food safety and natural disasters among currently married people: Multi-level logistic regression analysis (N = 3557). Appendix S3. Factors associated with concern about radiation, food safety and natural disaster among people aged at least 40: Multi-level logistic regression analysis (N = 4230). Appendix S4. Factors associated with concern about radiation, food safety and natural disasters among people with household incomes at least 3 million yen: Multi-level logistic regression analysis (N = 3691).
(DOC)
