# Supplementary material for: Who Was Concerned about Radiation, Food Safety, and Natural Disasters after the Great East Japan Earthquake and Fukushima Catastrophe? A Nationwide Cross-Sectional Survey in 2012
Source: PLoS One. 2014 Sep 2;9(9):e106377. doi: 10.1371/journal.pone.0106377 (PMC4152271; doi:10.1371/journal.pone.0106377)
Supplement: File S1 — This file contains Appendix S1–Appendix S4. Appendix S1. Factors associated with concern about radiation, food safety and natural disasters: Pooled inference of multi-level logistic regression analysis after multiple iterative regression imputation (N = 6451). Appendix S2. Factors associated with concern about radiation, food safety and natural disasters among currently married people: Multi-level logistic regression analysis (N = 3557). Appendix S3. Factors associated with concern about radiation, food safety and natural disaster among people aged at least 40: Multi-level logistic regression analysis (N = 4230). Appendix S4. Factors associated with concern about radiation, food safety and natural disasters among people with household incomes at least 3 million yen: Multi-level logistic regression analysis (N = 3691). (DOC) [file pone.0106377.s001.doc]

Appendix S1. Factors associated with concern about radiation, food safety and natural disasters: Pooled inference of multi-level logistic regression analysis after multiple iterative regression imputation (N = 6451).

|  | Concern about Radiation | | | | Concern about Food Safety | | | | Concern about Natural Disasters | | | |
| --- | --- | --- | --- | --- | --- | --- | --- | --- | --- | --- | --- | --- |
| Predictor variables | Crude OR (95% CI) | *p*-value | Adjusted OR (95% CI) | *p*-value | Crude OR (95% CI) | *p*-value | Adjusted OR (95% CI) | *p*-value | Crude OR (95% CI) | *p*-value | Adjusted OR (95% CI) | *p*-value |
| Gender (ref: man) | 1.70 (1.54–1.87) | < 0.001 | 1.67 (1.37–2.03) | < 0.001 | 1.88 (1.70–2.07) | < 0.001 | 1.75 (1.44–2.13) | < 0.001 | 1.84 (1.64–2.07) | < 0.001 | 1.84 (1.48–2.28) | < 0.001 |
| Parenthood (ref: without children) | 1.16 (1.03–1.31) | 0.01 | 0.93 (0.75–1.16) | 0.52 | 1.26 (1.12–1.42) | < 0.001 | 0.96 (0.77–1.19) | 0.72 | 0.97 (0.86–1.09) | 0.62 | 0.94 (0.74–1.19) | 0.62 |
| Care Needs (ref: no care needs) | 1.14 (0.97–1.33) | 0.10 | 1.26 (0.83–1.90) | 0.27 | 1.08 (0.93–1.27) | 0.32 | 1.27 (0.84–1.92) | 0.25 | 1.07 (0.92–1.25) | 0.38 | 1.23 (0.79–1.94) | 0.36 |
| Gender * Parenthood | 1.58 (1.44–1.75) | < 0.001 | 1.07 (0.85–1.36) | 0.56 | 1.82 (1.65–2.01) | < 0.001 | 1.09 (0.86–1.38) | 0.45 | 1.58 (1.41–1.78) | < 0.001 | 1.07 (0.83–1.38) | 0.59 |
| Gender * Care needs | 1.51 (1.21–1.87) | < 0.001 | 0.83 (0.45–1.52) | 0.54 | 1.60 (1.32–1.95) | < 0.001 | 0.73 (0.41–1.32) | 0.30 | 1.54 (1.22–1.94) | < 0.001 | 0.83 (0.43–1.58) | 0.56 |
| Care needs * Parenthood | 1.17 (0.98–1.40) | 0.08 | 0.86 (0.53–1.40) | 0.55 | 1.15 (0.96–1.37) | 0.12 | 0.68 (0.42–1.12) | 0.13 | 1.04 (0.86–1.27) | 0.69 | 0.82 (0.48–1.39) | 0.46 |
| Gender * Parenthood * Care needs | 1.60 (1.26–2.02) | < 0.001 | 1.30 (0.64–2.63) | 0.47 | 1.84 (1.45–2.33) | < 0.001 | 1.95 (0.97–3.96) | 0.06 | 1.55 (1.18–2.04) | 0.002 | 1.38 (0.64–2.96) | 0.41 |
| Age (10 years incr.) | 0.98 (0.96–1.00) | 0.046 | 0.92 (0.89–0.96) | < 0.001 | 0.97 (0.95–0.99) | 0.003 | 0.90 (0.87–0.94) | < 0.001 | 0.93 (0.91–0.95) | < 0.001 | 0.87 (0.84–0.90) | < 0.001 |
| Married | 1.35 (1.22–1.49) | < 0.001 | 1.49 (1.30–1.71) | < 0.001 | 1.35 (1.22–1.49) | < 0.001 | 1.40 (1.22–1.61) | < 0.001 | 1.11 (0.98–1.24) | 0.10 | 1.28 (1.12–1.47) | < 0.001 |
| Child cohabitation | 1.17 (1.06–1.29) | 0.001 | 0.97 (0.85–1.11) | 0.67 | 1.43 (1.30–1.58) | < 0.001 | 1.25 (1.09–1.43) | 0.002 | 1.12 (0.99–1.26) | 0.07 | 1.00 (0.85–1.17) | 1.00 |
| College Completion | 0.91 (0.80–1.05) | 0.20 | 0.99 (0.86–1.14) | 0.89 | 1.07 (0.95–1.21) | 0.24 | 1.28 (1.12–1.47) | < 0.001 | 0.97 (0.85–1.11) | 0.67 | 1.06 (0.91–1.24) | 0.45 |
| Household income (one level incr.)† | 1.01 (0.97–1.05) | 0.62 | 0.98 (0.94–1.02 | 0.32 | 1.00 (0.96–1.04) | 1.00 | 0.96 (0.92–1.00) | 0.046 | 1.01 (0.97–1.05) | 0.62 | 0.97 (0.93–1.01) | 0.13 |
| Student (%)ref: with job income | 0.68 (0.54–0.87) | 0.002 | 0.65 (0.49–0.86) | 0.002 | 0.74 (0.59–0.94) | 0.01 | 0.81 (0.62–1.07) | 0.13 | 0.84 (0.65–1.08) | 0.17 | 0.61 (0.45–0.81) | < 0.001 |
| Retired (%)ref: with job income | 0.94 (0.82–1.08) | 0.39 | 1.17 (0.98–1.40) | 0.08 | 0.85 (0.74–0.98) | 0.02 | 1.22 (1.02–1.46) | 0.03 | 0.86 (0.75–0.99) | 0.03 | 1.20 (1.00–1.43) | 0.046 |
| Homemaker (%) ref: with job income | 1.55 (1.33–1.82) | < 0.001 | 1.15 (0.95–1.40) | 0.16 | 1.82 (1.56–2.13) | < 0.001 | 1.38 (1.13–1.68) | 0.001 | 1.35 (1.13–1.61) | < 0.001 | 1.00 (0.81–1.24) | 1.00 |
| Other employment status (%)ref: with job income | 0.78 (0.64–0.95) | 0.01 | 0.82 (0.66–1.02) | 0.07 | 1.15 (0.95–1.40) | 0.16 | 1.30 (1.05–1.61) | 0.02 | 0.84 (0.67–1.04) | 0.10 | 0.86 (0.68–1.09) | 0.21 |
| House Damage Certificate | 1.60 (1.19–2.15) | 0.002 | 1.40 (1.05–1.89) | 0.02 | 1.28 (1.00–1.66) | 0.055 | 1.11 (0.84–1.45) | 0.48 | 1.40 (1.05–1.89) | 0.02 | 1.26 (0.92–1.72) | 0.15 |
| Current Evacuation | 2.83 (1.04–7.69) | 0.04 | 2.59 (0.93–7.16) | 0.07 | 1.26 (0.57–2.76) | 0.57 | 1.23 (0.55–2.76) | 0.61 | 2.41 (0.82–7.09) | 0.11 | 2.23 (0.76–6.54) | 0.15 |
| Tohoku-DRA region (ref: Other region) | 3.10 (1.69–5.68) | < 0.001 | 2.92 (2.01–4.23) | < 0.001 | 1.57 (1.10–2.23) | 0.01 | 1.51 (1.12–2.02) | 0.006 | 1.62 (0.97–2.69) | 0.06 | 1.45 (0.89–2.36) | 0.14 |
| Tohoku-not-DRA region (ref: Other region) | 0.90 (0.37–2.16) | 0.81 | 1.09 (0.78–1.53) | 0.60 | 0.84 (0.54–1.29) | 0.41 | 0.87 (0.66–1.14) | 0.32 | 0.66 (0.39–1.13) | 0.69 | 0.70 (0.45–1.11) | 0.13 |
| Kanto region (ref: Other region) | 1.80 (0.82–3.95) | < 0.001 | 2.18 (1.66–2.87) | < 0.001 | 1.27 (0.89–1.81) | 0.18 | 1.34 (1.12–1.59)) | 0.001 | 1.55 (1.18–2.04) | 0.002 | 1.40 (0.93–2.12) | 0.11 |

†: Range is level 0 indicating zero household income and level 7 indicating at least 10 million yen of that. OR: odds ratio. DRA: invoke of the disaster relief act. The fitted formula assumed linearity between an outcome with item-non-response (three concerns, parenthood, child cohabitation, marital status, family care needs, education, dummy variables for employment status and household income) and additive predictors, and no interaction term was constructed. The predictors were imputed variables (except for an outcome to be imputed) and fully observed variables (gender, age, the house-damage certificate, current evacuation status, the 10 areas, and the 4 regional indicator). Although some auxiliary variables were available from the dataset, they were not included in the fitted formula because convergence had hardly been achieved based on the Gelman-Rubin convergence statistic (R-hat). After convergence by 637 iterations, inferences were pooled across 3 datasets.

Appendix S2. Factors associated with concern about radiation, food safety and natural disasters among currently married people: Multi-level logistic regression analysis (N = 3557)

|  | Concern about Radiation | | Concern about Food Safety | | Concern about Natural Disaster | |
| --- | --- | --- | --- | --- | --- | --- |
| Predictor variables | Adjusted OR (95% CI) | *p*-value | Adjusted OR (95% CI) | *p*-value | Adjusted OR (95% CI) | *p*-value |
| Gender (ref: man) | 2.31 (1.40–3.83) | 0.001 | 2.31 (1.42–3.75) | < 0.001 | 1.59 (0.91–2.75) | 0.10 |
| Parenthood (ref: without children) | 1.00 (0.68–1.45) | 0.98 | 1.09 (0.74–1.59) | 0.67 | 0.81 (0.54–1.21) | 0.29 |
| Care Needs (ref: no care needs) | 0.63 (0.24–1.61) | 0.33 | 0.88 (0.34–2.24) | 0.78 | 1.06 (0.38–2.91) | 0.92 |
| Gender * Parenthood | 0.81 (0.48–1.38) | 0.44 | 0.89 (0.53–1.48) | 0.64 | 1.14 (0.64–2.02) | 0.67 |
| Gender * Care needs | 1.04 (0.26–4.20) | 0.96 | 0.87 (0.22–3.48) | 0.84 | 1.52 (0.29–8.02) | 0.62 |
| Care needs * Parenthood | 1.71 (0.64–4.59) | 0.28 | 1.00 (0.37–2.68) | 0.99 | 0.93 (0.32–2.68) | 0.90 |
| Gender * Parenthood * Care needs | 0.92 (0.21–3.93) | 0.91 | 1.62 (0.38–6.88) | 0.52 | 0.74 (0.13–4.18) | 0.74 |
| Age (10 years incr.) | 0.97 (0.92–1.03) | 0.30 | 0.89 (0.84–0.94) | < 0.001 | 0.91 (0.86–0.96) | 0.001 |
| Married | N.A. | N.A. | N.A. | N.A. | N.A. | N.A. |
| Child cohabitation | 1.04 (0.88–1.24) | 0.64 | 1.33 (1.12–1.58) | < 0.001 | 1.09 (0.91–1.31) | 0.37 |
| College Completion | 0.99 (0.82–1.18) | 0.88 | 1.32 (1.10–1.58) | 0.003 | 1.03 (0.85–1.24) | 0.80 |
| Household income (one level incr.)† | 1.00 (0.95–1.05) | 0.87 | 0.96 (0.91–1.01) | 0.11 | 0.97 (0.92–1.03) | 0.33 |
| House Damage Certificate | 1.59 (1.06–2.39) | 0.03 | 1.26 (0.88–1.78) | 0.20 | 1.16 (0.77–1.75) | 0.48 |
| Current Evacuation | 1.16 (0.28–4.88) | 0.83 | 1.11 (0.32–3.80) | 0.87 | 0.96 (0.24–3.84) | 0.96 |
| Tohoku-DRA region (ref: Other region) | 4.84 (2.96–7.92) | < 0.001 | 1.81 (1.19–2.74) | 0.006 | 2.19 (1.16–4.16) | 0.02 |
| Tohoku-not-DRA region (ref: Other region) | 1.24 (0.85–1.83) | 0.27 | 0.93 (0.63–1.38) | 0.74 | 0.75 (0.42–1.34) | 0.34 |
| Kanto region (ref: Other region) | 2.47 (1.92–3.19) | < 0.001 | 1.49 (1.15–1.93) | 0.003 | 1.56 (0.96–2.56) | 0.08 |

†: Range is level 0 indicating zero household income and level 7 indicating at least 10 million yen of that. OR: odds ratio. DRA: invoke of the disaster relief act. Dummy variables for employment status were not included because of a model-fitting problem, especially in the “student” category.

Appendix S3. Factors associated with concern about radiation, food safety and natural disaster among people aged at least 40: Multi-level logistic regression analysis (N = 4230)

|  | Adjusted OR (95% CI) | | |
| --- | --- | --- | --- |
| Predictor variables | Concern about Radiation | Concern about Food Safety | Concern about Natural Disaster |
| Gender (ref: man) | 1.94 (1.37–2.74) | 2.03 (1.44–2.85) | 1.99 (1.37–2.87) |
| Parenthood (ref: without) | 1.04 (0.78–1.39) | 1.04 (0.78–1.38) | 0.93 (0.69–1.24) |
| Care Needs (ref: not) | 1.85 (0.97–3.53) | 1.24 (0.67–2.31) | 1.33 (0.68–1.24) |
| Gender * Parenthood | 0.94 (0.64–1.37) | 0.97 (0.67–1.40) | 0.95 (0.63–1.42) |
| Gender * Care needs | 1.03 (0.39–2.73) | 0.90 (0.36–2.25) | 0.97 (0.33–2.82) |
| Care needs * Parenthood | 0.58 (0.29–1.17) | 0.73 (0.37–1.46) | 0.73 (0.35–1.51) |
| Gender * Parenthood * Care needs | 1.03 (0.36–2.94) | 1.49 (0.55–4.05) | 1.10 (0.35–3.46) |
| Age (10 years incr.) | 0.87 (0.81–0.94) | 0.79 (0.73–0.84) | 0.90 (0.85–0.95) |
| Married | 1.49 (1.27–1.76) | 1.21 (1.03–1.43) | 1.30 (1.09–1.54) |
| Child cohabitation | 0.96 (0.83–1.12) | 1.22 (1.05–1.42) | 1.01 (0.86–1.28) |
| College Completion | 0.96 (0.81–1.15) | 1.27 (1.06–1.51) | 1.02 (0.85–1.18) |
| Household income (one level incr.)† | 0.99 (0.95–1.04) | 0.99 (0.94–1.03) | 0.98 (0.93–1.03) |
| Student (%)ref: with job income | 1.09 (0.07–17.6) | 0.91 (0.06–14.7) | N.A. |
| Retired (%)ref: with job income | 1.34 (1.09–1.65) | 1.60 (1.30–1.96) | N.A. |
| Homemaker (%) ref: with job income | 0.90 (0.73–1.12) | 1.14 (0.92–1.41) | N.A. |
| Other employment status (%)ref: with job income | 0.77 (0.59–1.01) | 1.01 (0.78–1.32) | N.A. |
| House Damage Certificate | 1.70 (1.16–2.49) | 1.25 (0.90–1.75) | 1.43 (0.96–2.11) |
| Current Evacuation | 1.55 (0.48–5.03) | 1.03 (0.39–2.75) | 2.68 (0.59–12.10) |
| Tohoku-DRA region (ref: Other region) | 3.82 (2.40–6.06) | 1.59 (1.15–2.19) | 1.63 (0.94–2.83) |
| Tohoku-not-DRA region (ref: Other region) | 1.26 (0.83–1.90) | 0.84 (0.62–1.15) | 0.75 (0.45–1.26) |
| Kanto region (ref: Other region) | 2.33 (1.69–3.21) | 1.37 (1.17–1.62) | 1.35 (0.87–2.09) |

†: Range is level 0 indicating zero household income and level 7 indicating at least 10 million yen of that. OR: odds ratio. DRA: invoke of the disaster relief act. Dummy variables for employment status were not included about natural disaster because of a model-fitting problem in the “student” category.

Appendix S4. Factors associated with concern about radiation, food safety and natural disasters among people with household incomes at least 3 million yen: Multi-level logistic regression analysis (N = 3691)

|  | Adjusted OR (95% CI) | | |
| --- | --- | --- | --- |
| Predictor variables | Concern about Radiation | Concern about Food Safety | Concern about Natural Disaster |
| Gender (ref: man) | 1.74 (1.33–2.27) | 1.75 (1.34–2.27) | 1.90 (1.43–2.54) |
| Parenthood (ref: without children) | 0.93 (0.67–1.28) | 1.23 (0.89–1.69) | 0.97 (0.69–1.35) |
| Care Needs (ref: no care needs) | 0.82 (0.47–1.45) | 0.95 (0.54–1.69) | 1.84 (0.98–3.47) |
| Gender * Parenthood | 1.10 (0.80–1.53) | 1.11 (0.80–1.53) | 1.07 (0.75–1.53) |
| Gender * Care needs | 1.04 (0.48–2.26) | 1.02 (0.47–2.23) | 0.53 (0.22–1.28) |
| Care needs * Parenthood | 1.23 (0.64–2.37) | 0.85 (0.43–1.66) | 0.45 (0.22–0.92) |
| Gender * Parenthood * Care needs | 0.94 (0.38–2.33) | 1.53 (0.62–3.82) | 2.17 (0.79–5.98) |
| Age (10 years incr.) | 0.95 (0.90–1.01) | 0.88 (0.83–0.94) | 0.88 (0.82–0.94) |
| Married | 1.50 (1.22–1.84) | 1.32 (1.08–1.62) | 1.31 (1.05–1.63) |
| Child cohabitation | 0.97 (0.80–1.18) | 1.17 (0.97–1.42) | 1.01 (0.82–1.23) |
| College Completion | 0.92 (0.79–1.09) | 1.20 (1.02–1.41) | 1.05 (0.88–1.25) |
| Household income (one level incr.)† | 1.03 (0.96–1.11) | 0.99 (0.92–1.06) | 0.97 (0.90–1.05) |
| Student (%)ref: with job income | 0.62 (0.44–0.89) | 0.81 (0.57–1.16) | 0.58 (0.40–0.84) |
| Retired (%)ref: with job income | 1.15 (0.88–1.51) | 1.38 (1.05–1.80) | 1.29 (0.97–1.71) |
| Homemaker (%) ref: with job income | 1.12 (0.86–1.46) | 1.25 (0.97–1.61) | 0.87 (0.65–1.16) |
| Other employment status (%)ref: with job income | 0.72 (0.51–1.01) | 1.21 (0.86–1.70) | 0.81 (0.56–1.18) |
| House Damage Certificate | 1.46 (0.99–2.16) | 1.20 (0.85–1.69) | 1.19 (0.79–1.79) |
| Current Evacuation | 1.89 (0.49–7.28) | 0.71 (0.23–2.16) | 0.82 (0.25–2.74) |
| Tohoku-DRA region (ref: Other region) | 4.08 (2.65–6.28) | 1.48 (0.99–2.21) | 1.89 (1.05–3.41) |
| Tohoku-not-DRA region (ref: Other region) | 1.10 (0.78–1.55) | 0.76 (0.52–1.12) | 0.78 (0.46–1.33) |
| Kanto region (ref: Other region) | 2.16 (1.85–2.54) | 1.32 (1.05–1.66) | 1.52 (0.99–2.34) |

†: Range is level 4 indicating 3-3.9 million yen of household income and level 7 indicating at least 10 million yen of that. OR: odds ratio. DRA: invoke of the disaster relief act.
